# Time-weighted urine oxygen tension as a predictor of acute kidney injury in patients with sepsis: a preliminary prospective observational study

**DOI:** 10.1080/0886022X.2025.2575441

**Published:** 2025-11-02

**Authors:** Jiangtao Li, Fangqin Han, Yuqiu Lu, Ying Zhou, Lirui Wang, Jie Gao

**Affiliations:** a Department of Nephrology, Tongji University School of Medicine, Tongji Hospital, Shanghai, China; b Department of Intensive Care Unit, Nanxiang Hospital of Jiading District, Shanghai, China

**Keywords:** Acute kidney injury, sepsis, Kidney Disease Improving Global Outcomes classification, urine oxygen tension, time-weighted average

## Abstract

**Background:**

Septic associated acute kidney injury (SA-AKI) is common in the critically ill. Inadequate renal medullary tissue oxygenation has been linked to its pathogenesis. The aim of this preliminary study was to assess the feasibility of intermittent PuO_2_ monitoring using a blood gas analyzer in sepsis patients; to explore the effectiveness of time-weighted average PuO_2_ (PuO_2TW_) for predicting SA-AKI.

**Methods:**

A total of 76 consecutive adult patients who were admitted to our intensive care unit (ICU) from September 2023 to March 2024 were prospectively recruited. PuO_2_ was measured with a blood gas analyzer at 0h, 3h, and 6h after ICU admission. PuO_2TW_ was determined by the sum of the mean PuO_2_ values among consecutive time points multiplied by the period of time between consecutive time points and then dividing by the total time. All patients were followed throughout the ICU stay, and the development of SA-AKI during 48 h was evaluated.

**Results:**

Approximately 23.68% developed AKI during the ICU stay. PuO_2TW_ was lower in patients who developed AKI. The ROC curve analysis revealed that lower PuO_2TW_ was associated with AKI development at the cutoff of <68 mmHg (area under the curve [AUC] 0.687; *p* = .008). In the logistic regression models, PuO_2TW_ lower than 68 mmHg was associated with the development of AKI, when adjusted by confounding factors (OR 8.20; *p* = .002).

**Conclusions:**

Measurement of PuO_2_ is feasible by collecting urine from a Foley catheter for analysis in a blood gas machine. 6h PuO_2TW_ had a significant independent predictive value for AKI.

## Introduction

Sepsis-associated acute kidney injury (SA-AKI) is a frequently occurring complication of the critically ill patients in intensive care unit (ICU) and contributes to unacceptable high rates of morbidity and mortality [1,2]. Detection of septic patients at risk of AKI is critical for timely and adequate supportive care. Current definition of AKI is based on both increases in serum creatinine levels and decreases in urine output [3]. Unfortunately, these monitoring methods can lead to delayed diagnosis and ineffective interventions [4,5]. Therefore, the identification of other reliable and cost-effective biomarkers to predict SA-AKI earlier than the current gold standard is of great clinical value.

One of the mechanisms implicated in the pathophysiology of SA-AKI is hypoxia of renal tissue, particularly in the renal medulla [6]. When AKI is induced by *E. coli* resulting in reduced creatinine clearance or oliguria, a decline of oxygenation in renal medullary tissue is observed [7]. In the ovine septic AKI model, medullary tissue hypoxia precedes the occurrence of functional deficits by several hours [8]. Taken together, these preclinical observations indicate that medullary hypoxia may be a potential biomarker for early detection of impending AKI, providing an opportunity to avoid its development.

Evidence from both animal and clinical studies supports the view that non-invasively measured bladder urinary oxygen tension (PuO_2_) can provide a reliable estimate of oxygen tension in renal medulla, which can only be measured invasively [9]. Bladder PuO_2_ can be measured continuously using a fiber optic probe, polarographic electrode or magnetic resonance imaging [10–12]. Technical availability issues, measurement restrictions and practical reasons, however, are challenges to overcome for these approaches to be routinely used in clinics.

Urinary PO_2_ can also be measured intermittently by collecting urine from a Foley catheter for analysis in a blood gas analyzer [13–15]. This approach, however, cannot reflect the duration of PuO_2_ decrease, and thus is considered inferior to the diagnostic power of continuous PuO_2_ monitoring. Time-weighted averaging approach is an increasingly utilized method that takes into consideration not only the numerical levels of a particular variable, but also the amount of time spent on it [16,17]. We assume that time-weighted average PuO_2_ (PuO_2TW_), which derived from serial PuO_2_ measurements, would more accurately reflect the impact of cumulative PuO_2_ decrease on renal outcome. The aim of this preliminary study, therefore, was to assess the feasibility of intermittent PuO_2_ monitoring using a blood gas analyzer in sepsis patients; to explore the effectiveness of PuO_2TW_ for predicting SA-AKI.

## Methods

### Study design, patients and study size

A single-center, prospective observational study was conducted in accordance with Good Clinical Practice and the ethical principles outlined in the Declaration of Helsinki. The study was approved by the Ethics Committee of Nanxiang Hospital of Jiading District. (Authorization number: 2022013) and written informed consent was obtained from all patients or relatives prior to their inclusion in the study.

Consecutive adult patients who were admitted to our intensive care unit (ICU) from September 2023 to March 2024 were prospectively recruited. The inclusion criteria for the patients were as follows: (1) willing to participate in the study; (2) were ≥18 years of age; (3) fulfilled the criteria for sepsis-3 [18]. Exclusion criteria were: (1) anticipated ICU stay < 48 h; (2) existing AKI before admission to the ICU; (3) end-stage kidney disease (ESKD) or stage 5 chronic kidney disease; (4) history of urinary tract modification surgery; (5) pregnant women. Due to the exploratory nature of the study and the difficulty of establishing a sample size using statistical methodology, the target number of patients was tentatively set at 50.

### Clinical endpoint and definitions

The primary endpoint was the development of AKI within 48 h after ICU admission. According to the most recent sepsis consensus, this syndrome is defined as the presence of an infection combined with an acute change in the ‘Sequential Organ Failure Assessment’ (SOFA) score of two points or more [18].

AKI was defined according to Kidney Disease Improving Global Outcomes (KDIGO) criteria [3], using the increase in serum creatinine ≥0.3 mg/dL within 48 h; or increase in serum creatinine to ≥1.5 times baseline within seven days. Because most patients had the catheter removed 6h after urine collection, we did not utilize the urine output criterion for diagnosis of AKI. Baseline kidney function was defined as the most recent outpatient, non-emergency department serum creatinine concentration between 7 and 365 days before sepsis diagnosis. If no previous results were available, baseline creatinine was the lowest creatinine value between that calculated from an estimated glomerular filtration rate (eGFR) of 75 mL/min/1.73 m^2^ and creatinine reported on admission.

### Data collection and biomarkers measurement

At the time of the patients’ enrollment, demographic information, the Acute Physiology and Chronic Health Evaluation (APACHE II) score and the Sequential Organ Failure Assessment (SOFA) score were recorded. Urine samples were taken at 0 h, 3 h, and 6 h after admission to measure PuO_2_. All patients were followed during their ICU stay, and the development of AKI was evaluated.

### Measurement of PuO_2_

After ICU admission, a urinary catheter (16 F Foley catheter) was inserted. The catheter was connected to a system of standard urine collection. Urine samples were anaerobically collected from a urine sampling port using a 1-mL syringe with a needle. In the aspiration process, application a negative pressure during suctioning may cause the compression of the catheter. The PuO_2_ of urine entering the bladder from the ureter would contaminate urine sample. To overcome this technical problem, the proximal end of the catheter was clamped prior to aspiration, ensuring no oxygen-contaminated urine from ureter was collected. Silicone is known for its high oxygen permeability, and probably caused substantial oxygenation of the urine as it passed through the flush lumen, resulting in the increased PuO_2_ measurement. We tried suctioning gently to lower the aspiration pressure to control this confounder. In our study, PuO_2_ was measured with a blood gas analyzer. Of note, the BGA was located inside the unit and measurements were conducted as fast as possible. The procedure of urine collection and PuO_2_ measurements were undertaken by research-trained nurses.

### Calculation of PuO_2TW_

PuO_2TW_ was determined by the sum of the mean PuO_2_ values among consecutive time points multiplied by the period of time between consecutive time points and then dividing by the total time.

### Statistical analysis

Data are expressed as the mean ± SD, the median (including the lower and upper quartiles) or percentage. Comparisons between two groups for continuous variables were made using Student’s t test or the Mann–Whitney U test. Comparisons between two groups for categorical variables were made using the *χ*^2^ test or Fisher’s exact test. Receiver operating characteristic (ROC) curve analysis was performed to determine the performance of PuO2_TW_ to predict AKI development. To analyze the independent association of PuO_2TW_ with different parameters, a multivariate linear regression analysis was performed. The variables introduced into the model were those that reached statistical significance in the univariate analysis. Sensitivity analysis was performed by excluding patients who had a prior history of CKD. Data analysis was performed using IBM SPSS Statistics for Windows (version 28.0. Armonk, NY: IBM Corp) and Prism v.8 (Graph Pad Software). *p* Values lower than.05 were considered statistically significant.

## Results

**Figure 1.**
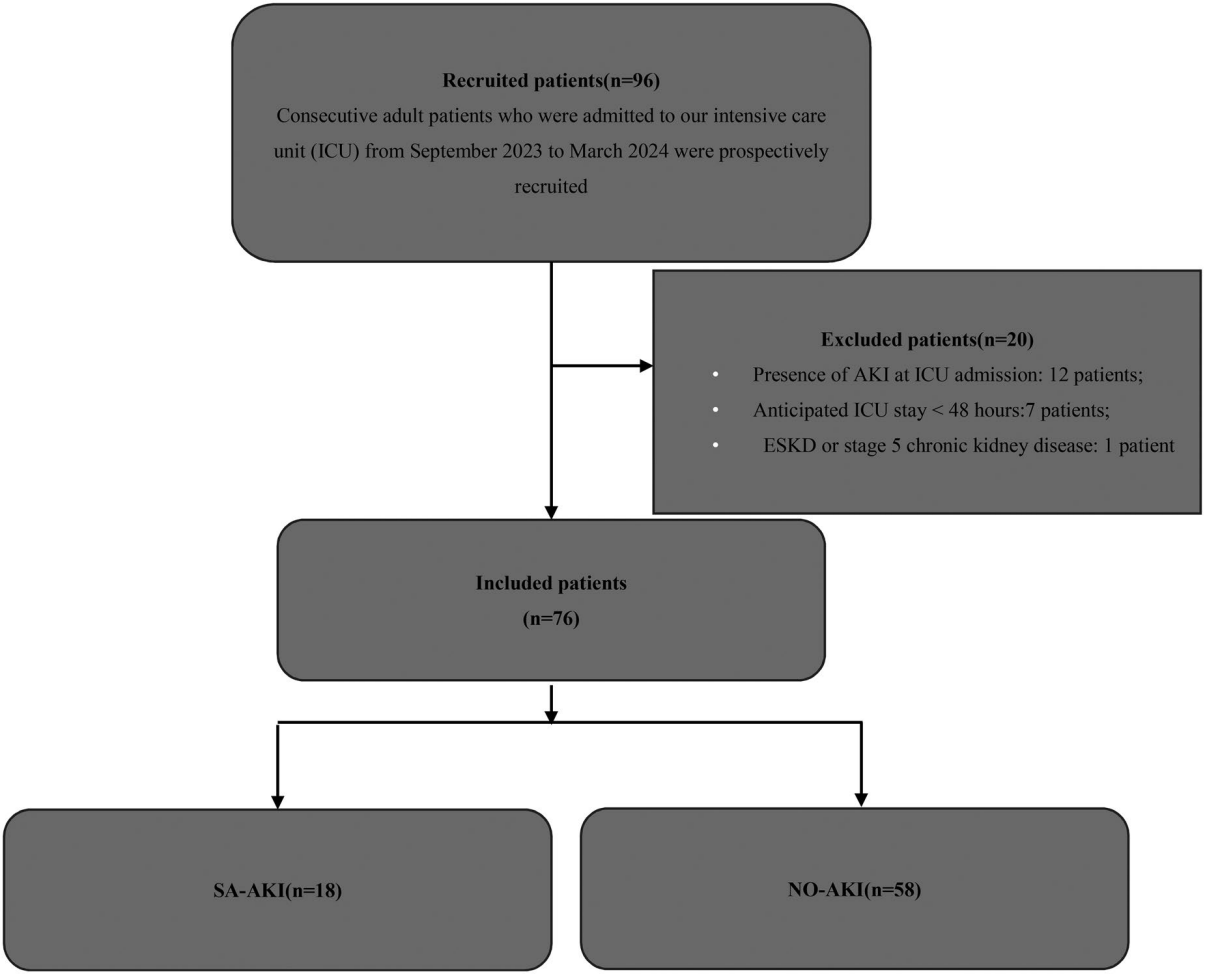
Flow chart for study inclusion/exclusion of patients. AKI: acute kidney injury; ICU: intensive care unit; ESKD: end-stage kidney disease; SA-AKI: septic associated acute kidney injury; NO-AKI: patients who did not develop acute kidney injury.

During the study, 96 consecutive patients were admitted with a diagnosis of sepsis in the ICU; however, 20 patients were excluded (presence of AKI at ICU admission: 12 patients; anticipated ICU stay < 48 h: 7 patients; ESKD or stage 5 chronic kidney disease: 1 patient). Thus, we evaluated 76 patients (Figure 1). The mean age was 77 (70–87) years, 57.89% were male and the median length of ICU stay was 14 days (5.5–20.5). The mortality rate during the ICU stay was 15.79%.

The demographic and clinical data are presented in Table 1. Among those patients with sepsis, 23.68% developed AKI during the ICU stay. Regarding KDIGO stages, 66.8% were classified as KDIGO 1, 16.6% as KDIGO 2 and 16.6% as KDIGO 3. Among patients who developed AKI, 16.6% needed dialysis during the ICU stay. Patients with AKI tended to have higher APACH II and SOFA scores. In addition, patients with AKI had a high rate of CKD and mortality.

**Table 1.** Demographic and clinical data of 76 patients with sepsis.

	Acute kidney injury		*p* Value
Variable	Yes (*n* = 18)	No (*n* = 58)	
Age (years)	73.0 (56.0,83.0)	78.0 (73.0,87.3)	.03
Male sex, *n* (%)	15 (51.7)	29.0 (61.7)	.48
APACHE II score	18.7 ± 5.2	16.8 ± 5.1	.19
SOFA score	6.0 (3.0,10.0)	6.0 (3.0,8.0)	.75
Sepsis source, *n* (%)			.08
Respiratory	9 (50.0)	42 (72.4)	
Abdominal	7 (38.9)	12 (20.7)	
Urinary	0 (0)	3 (5.2)	
Others	2 (11.1)	1 (1.7)	
CKD, *n* (%)	7 (38.9)	6 (10.3)	<.01
MV, *n* (%)	4 (22.2)	15 (25.9)	1.00
AKI stage, *n* (%)			
Stage 1	12 (66.8)		
Stage 2	3 (16.6)		
Stage 3	3 (16.6)		
Dialysis	3 (16.6)		
Norepinephrine, *n* (%) (within 6 h after ICU admission)	8 (44.4)	18 (31.0)	.39
Furosemide, *n* (%) (within 6 h after ICU admission)	0 (0)	0 (0)	1.00
Length of ICU stay (days)	13.5 (9.0,18.0)	15.0 (4.8,21.3)	.87
ICU mortality, *n* (%)	6.0 (33.3)	6.0 (10.3)	.03

*Notes:* Data are expressed as the mean ± *SD*, median (including the lower and upper quartiles) or percentage. APACHE II: Acute Physiology and Chronic Health Evaluation; SOFA: Sequential Organ Failure Assessment; CKD: chronic kidney disease; MV: mechanical ventilation; ICU: intensive care unit; AKI: acute kidney injury.

**Figure 2.**
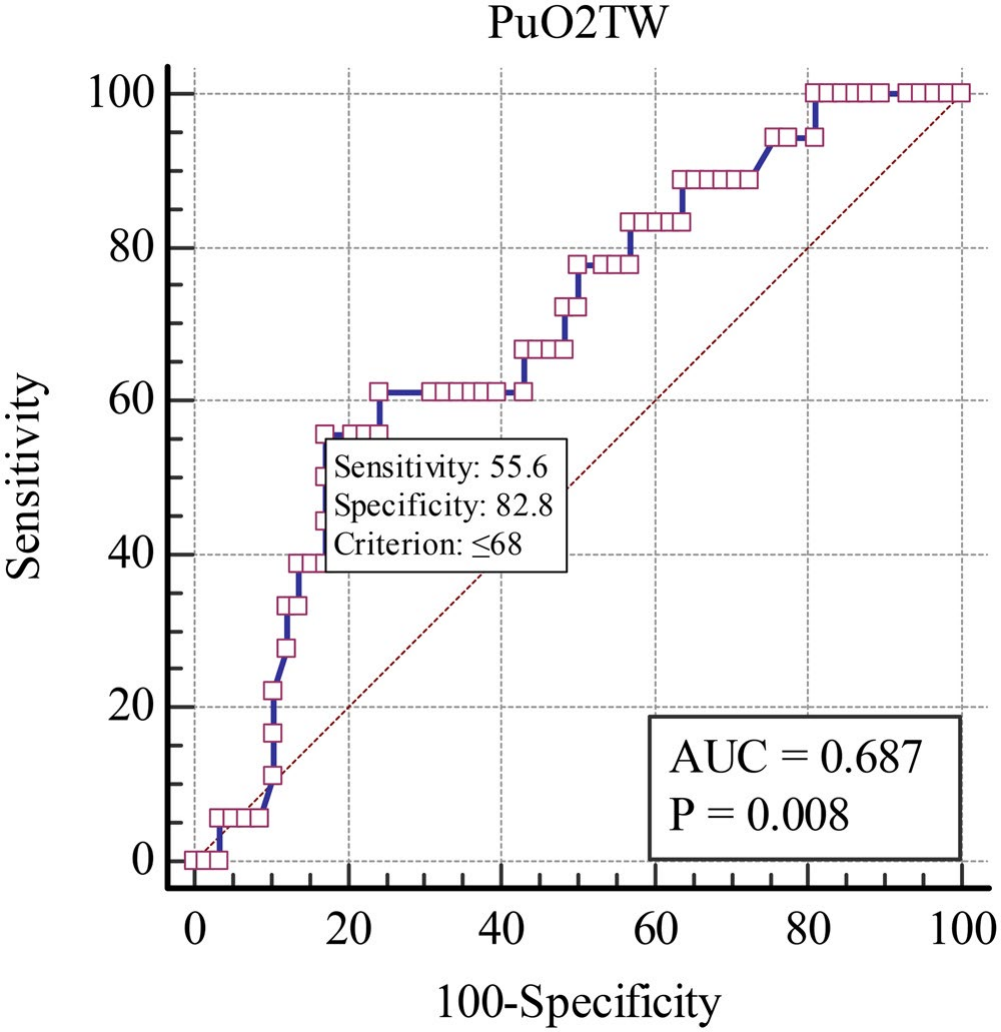
ROC curve for the association between PuO_2TW_ and AKI development. PuO2_TW_: time-weighted average PuO_2_; AUC: area under the curve.

A total of 228 PuO_2_ values were collected (3 values at each of the three time points), with a mean value 82.47 ± 30.64 mmHg. The laboratory data are presented in Table 2. PuO_2TW_ was lower in patients who developed AKI [non-AKI: 84.7 ± 21.8 vs AKI: 71.3 ± 18.0 mmHg; *p* = .02]. There were no differences between groups in the other laboratory data. The ROC curve analysis revealed that lower PuO2_TW_ was associated with AKI development (area under the curve [AUC] 0.687; CI 95% 0.587–0.802; *p* = .008) at the cutoff of <68mmHg [sensibility: 55.6% (CI 95% 30.8–78.5%); specificity: 82.8% (CI 95% 72.6–92.7%); positive predictive value: 52.6% (CI 95% 34.9–69.7%); negative predictive value: 86% (CI 95% 78.3–91.2%)] (Figure 2).

**Table 2.** Laboratory data of 76 patients with sepsis.

	Acute kidney injury		*p* Value
Variable	Yes (*n* = 18)	No (*n* = 58)	
Hemoglobin (g/dL)	10.9 ± 2.9	10.9 ± 2.5	.99
Hematocrit (%)	29.6 (25.8,34.7)	32.0 (29.1,39.7)	.33
Leukocytes (10^3^/mm^3^)	16.1 (12.3,20.5)	15.9 (15.6,18.3)	.79
CRP (mg/dL)	125.5 (113.9,187.3)	95.8 (54.3,164.1)	.13
PCT (ng/mL)	12.6 (10.2,15.2)	4.9 (1.4,27.9)	.21
Lactate (mmol/L)	2.9(1.7,8.3)	2.6 (1.6,4.1)	.46
Sodium (mmol/L)	140.5 (139.0,147.0)	140.0 (136.7,146.3)	.30
Potassium (mmol/L)	4.3 (3.3,4.5)	3.9 (4.1,4.5)	.29
Albumin (g/dL)	2.9 (2.6,3.0)	2.9 (2.7,3.1)	.42
Urea (mg/dL)	193.5 (91.8,379.8)	165.6 (133.2,278.5)	.98
Creatinine (mg/dL)	1.1 ± 0.3	1.0 ± 0.5	.32
MAP (mmHg)	83.9 ± 22.5	93.7 ± 21.0	.10
Mean urine flow (mL/min)	1.5 (1.3,1.8)	1.8 (1.1,2.3)	.11
Nadir PaO_2_ (mmHg)	102.2 ± 42.2	98.9 ± 35.1	.75
PuO_2_ (mmHg)			
T0	69.2 ± 31.2	85.1 ± 4.8	.10
T1	69.3 ± 24.0	81.3 ± 29.4	.12
T2	80.1 ± 33.2	89.7 ± 23.7	.23
PuO2_TW_ (mmHg)	71.3 ± 18.0	84.7 ± 21.8	.02

*Notes:* Data are expressed as the mean ± *SD*, median (including the lower and upper quartiles). CRP: C-reactive protein; PCT: procalcitonin; MAP: mean arterial pressure; PaO_2_: partial pressure of oxygen; urinary oxygen tension PuO_2_: urinary oxygen tension; PuO2_TW_: time-weighted average PuO_2_.

In the logistic regression models, PuO_2TW_ lower than 68 mmHg was associated with development of AKI when adjusted by age, gender, APACHE II, CKD, MAP, norepinephrine use and PaO_2_ (OR 8.20; CI 95% 2.12–31.67; *p* = .002, Table 3). Further logistic regression was performed after excluding CKD patients to strengthen the robustness of the findings. The results showed that PuO_2TW_ remained significantly associated with higher odds of developing AKI in the non-CKD group (OR 7.00; 95% CI 1.71–28.64, *p* = .007).

**Table 3.** Logistic regression analysis for the prediction of acute kidney injury in 76 patients with sepsis.

	Univariate analysis		Multivariable analysis	
Variable	OR (95%CI)	*p* Value	OR (95%CI)	*p* Value
Age	0.986 (0.948–1.024)	.461		
Sex	0.657 (0.227–1.903)	.439		
APACH II score	1.003 (0.898–1.119)	.962		
CKD	3.977 (1.190–13.288)	.025	7.501(1.789–31.444)	.006
MAP	0.984 (0.959–1.010)	.230		
Norepinephrine	1.778 (0.602–5.253)	.298		
Nadir PaO2	1.002 (0.988–1.017)	.763		
PuO2_TW_	4.800 (1.523–15.129)	.007	8.201(2.124–31.665)	.002

*Note:* CKD: chronic kidney disease; MAP: mean arterial pressure; PuO2TW: time-weighted average PuO2; APACHE II: Acute Physiology and Chronic Health Evaluation.

## Discussion

We performed a pilot prospective cohort study to assess the feasibility of intermittent PuO_2_ monitoring using a blood gas analyzer in septic patients; to explore the effectiveness of static and dynamic of PuO_2_ monitoring for predicting SA-AKI. We found that PuO_2_ measurement using a gas analyzer was feasible in septic patients. Moreover, we found that serial evaluation of PuO_2_ may be more helpful than a single point measurement, and dynamic changes of PuO_2_ may represent a useful monitoring tool of response to treatment. In this regard, PuO2_TW_ over the first 6 h in the ICU demonstrated to be a good marker to predict SA-AKI.

In current literature, PuO_2_ is measured with highly elaborate and sophisticated measures such as polarographic electrodes, fiber-optic probes, or magnetic resonance imaging [10–12]. Due to technical availability issues, we choose to measure PuO_2_ using a point-of-care standard blood gas analyzer which was on-site. In fact, this approach had been reported previously. Chalikias et al. found that both PuO_2_ post-percutaneous coronary intervention (PCI) and Δchange in post PCI-baseline levels were predictive of contrast-induced AKI [13]. In another study, measurement of PuO_2_ using a blood gas analyzer 6 h after admission to ICU had modest discrimination in detecting patients who later developed AKI [14]. In our study, however, the predictive power of a single point measurement of PuO_2_ for AKI was insufficient. The most parsimonious interpretation of our current findings is that our cohort was heterogeneous, having different admission diagnoses and different degrees of severity in critical illness. While in a single/specific diagnostic grouping (cardiac surgery/PCI), the timing of kidney injury is known, patients who are admitted to the ICU may be at any point of the hypothetical ‘AKI timeline’, which may range from exposure to renal injury to irreversible kidney damage. In this context, a single-point measurement may not able to mirror the complex dynamic of kidney injury.

Since single-point measurement failed to capture the fluctuating nature of sepsis, we employed a longitudinal approach using time-weighted averages to monitor PuO_2_. This methodology enabled us to track changes in PuO_2_ over time and its correlations with patient outcomes, offering a more detailed view of PuO_2_ decrease burden. Multiple studies have shown that the first 6 h of sepsis management are especially important from a diagnostic, pathogenic, and therapeutic perspective, and that steps taken during this period can have a significant impact on outcome [19–21]. We found that 6h PuO_2TW_ had a significant independent predictive value for AKI. Our findings suggest that serial evaluation of PuO_2_ may be more helpful than a single value, and PuO_2TW_ may represent a useful tool of AKI prediction.

SA-AKI is a heterogenous syndrome with multiple mechanisms [22]. Moreover, sepsis associated factors such as use of nephrotoxic medications or associated complications such as fluid overload can further contribute to AKI in patients with sepsis. Our findings imply that, in critically ill patients with sepsis, predicting AKI solely based on a single-point measurement of PuO_2_ without considering duration of PuO_2_ decrease, is an oversimplification, as it overlooks the complexities of this heterogenous syndrome. However, it is possible to estimate the burden of PuO_2_ decrease by calculating PuO_2TW_, especially during the first 6 h of ICU admission, and this can be done worldwide using blood gas analyzers.

In present study, several technical challenges remain for the measurement of PuO_2_. First, we collected urine samples from a Foley catheter. When measured in this way there is the potential for oxygen to diffuse from the atmosphere to the urine sample. To overcome this problem, urine samples were collected in a way that would minimize the opportunity for diffusion of oxygen from potential sources. PuO_2_ measured in our study was lower 82.47 ± 30.64 mmHg than that reported from the study (120–130 mmHg) in which urine samples were collected without confounders control [14]. Thus, it is reasonable to think that diffusion of oxygen from potential sources was partially controlled. Of note, PuO_2_ is not constant unless the urine volume is >6 mL/min [14]. Urine flow was 1.5–1.8 mL/min in our study. During low flow, urine in the catheter could be subject to the ingress of oxygen from the surrounding tissue. Therefore, the increase in PuO_2_ could not be completely avoided. Second, a prolonged time interval between sample collection and measurement can increase the opportunity of oxygen contamination. To control this confounder, measurements were conducted as fast as possible using a BGA situated in the unit. Finally, we measured PuO_2_ by a BGA. Because urine is outside the intended scope of the BGA, measurement of PuO_2_ using BGA requires validation or calibration. Currently, however, there is no golden standard for PuO_2_ measurement. Consequently, no methods-based approach can be used for technical validation or calibration. From a theoretical stand, since BGA is based on simple polarographic electrodes, it could be used to analyze other fluids aside from blood to PO_2_. Indeed, there is an increasing body of literature supporting the use of a BGA for PuO_2_ measurement [13–15].

In addition to the technical challenges, some other limitations need to be accounted for in this study. First, this was a single-center experience with a relatively small sample size. Therefore, bias could not be avoided. Patients who developed AKI were significantly younger than those who did not. This is counterintuitive, as advanced age is generally a known risk factor for AKI. This could be attributed to the exclusion of patients with anticipated ICU stay < 48 h and preexisting AKI. According to this exclusion criterion, most excluded patients were elderly. The small sample size also precluded a stratified analysis or subgroup assessments based on sepsis sources, severity, or other relevant variables. Furthermore, this was the first time time-weighted average was used to investigate the relationship between PuO_2_ and AKI. No established threshold exists for PuO_2TW_ and this small pilot study was not powered for diagnostic validation. Finally, the rate of AKI was low. The use of urine output criteria to identify additional patients who may have developed AKI could have added additional valuable data.

## Conclusions

Measurements of PuO_2_ are feasible by collecting urine from a Foley catheter for analysis in a blood gas machine. 6h PuO2_TW_ had a significant independent predictive value for AKI. Further studies are needed to validate this index and to elucidate whether PuO_2TW_ can be used to trigger interventions to successfully prevent or reduce the severity of AKI.

## Data Availability

The data sets used and/or analyzed during the current study are available from the corresponding author on reasonable request.
